# Intracellular removal of acetyl, feruloyl and *p*-coumaroyl decorations on arabinoxylo-oligosaccharides imported from lignocellulosic biomass degradation by *Ruminiclostridium cellulolyticum*

**DOI:** 10.1186/s12934-024-02423-z

**Published:** 2024-05-24

**Authors:** Nian Liu, Elise Odinot, Hélène David, Nicolas Vita, Felipe Mejia Otalvaro, Goetz Parsiegla, Yann Denis, Craig Faulds, Henri-Pierre Fierobe, Stéphanie Perret

**Affiliations:** 1 Aix-Marseille Université, CNRS, LCB-UMR7283, Marseille, France; 2 OléoInnov, 19 rue du Musée, Marseille, 13001 France; 3 Technical University of Denmark, The Novo Nordisk Foundation Center for Biosustainability, Konges Lyngby, 2800 Denmark; 4 Aix-Marseille Université, CNRS, BIP-UMR7281, Marseille, France; 5 Aix-Marseille Université, CNRS, IMM, Marseille, France; 6 Biodiversité et Biotechnologie Fongiques, INRAE, Aix Marseille University, Marseille, UMR1163, 13009 France

**Keywords:** Feruloyl esterase, *p*-coumaroyl esterase, Arabinoxylo-oligosaccharide, Arabinoxylan degradation

## Abstract

**Background:**

Xylans are polysaccharides that are naturally abundant in agricultural by-products, such as cereal brans and straws. Microbial degradation of arabinoxylan is facilitated by extracellular esterases that remove acetyl, feruloyl, and *p*-coumaroyl decorations. The bacterium *Ruminiclostridium cellulolyticum* possesses the Xua (xylan utilization associated) system, which is responsible for importing and intracellularly degrading arabinoxylodextrins. This system includes an arabinoxylodextrins importer, four intracellular glycosyl hydrolases, and two intracellular esterases, XuaH and XuaJ which are encoded at the end of the gene cluster.

**Results:**

Genetic studies demonstrate that the genes *xuaH* and *xuaJ* are part of the *xua* operon, which covers *xuaABCDD’EFGHIJ*. This operon forms a functional unit regulated by the two-component system XuaSR. The esterases encoded at the end of the cluster have been further characterized: XuaJ is an acetyl esterase active on model substrates, while XuaH is a xylan feruloyl- and *p*-coumaryl-esterase. This latter is active on oligosaccharides derived from wheat bran and wheat straw. Modelling studies indicate that XuaH has the potential to interact with arabinoxylobiose acylated with mono- or diferulate. The intracellular esterases XuaH and XuaJ are believed to allow the cell to fully utilize the complex acylated arabinoxylo-dextrins imported into the cytoplasm during growth on wheat bran or straw.

**Conclusions:**

This study reports for the first time that a cytosolic feruloyl esterase is part of an intracellular arabinoxylo-dextrin import and degradation system, completing its cytosolic enzymatic arsenal. This system represents a new pathway for processing highly-decorated arabinoxylo-dextrins, which could provide a competitive advantage to the cell and may have interesting biotechnological applications.

**Supplementary Information:**

The online version contains supplementary material available at 10.1186/s12934-024-02423-z.

## Introduction

Xylan is the main polysaccharide constituent of hemicellulose in agricultural by-products such as wheat bran and wheat straw. Xylan acts as a barrier for the direct hydrolysis of cellulose but is also itself a source of carbon and energy for xylanolytic organisms. Hence, its degradation increases the overall efficiency of converting lignocellulosic biomass into biofuel or other valuable chemicals [1]. In addition, xylan is considered as a prebiotic dietary fiber and its degradation in the gut to xylo-oligosaccharides contributes to a balanced healthy microbiota [2–4]. Therefore, acquiring further knowledge and understanding of the deconstruction and microbial utilization of xylan and xylo-oligosaccharides is of great importance for many biotechnological applications.

Xylan forms an heterogenous group of polysaccharides mainly composed of C-5 monosaccharides. They have a backbone of β-1,4 linked D-xylopyranosyl units substituted with α-1,2 and/or α-1,3 L-arabinofuranosyl (Ara*f*) residues and/ or α-1,2 (4-*O*-methyl) D-glucuronic acid. Depending on the nature of their decorations different sort of xylan can be distinguished, arabinoxylan (AX), glucuronoarabinoxylan (GAX), arabinoglucuronoxylan (AGX) and glucuronoxylan (GX) [5–7]. In addition to carbohydrate decorations, xylosyl units can be further acetylated at the *O*-2 and/or *O*-3 positions. In the same way, Ara*f* substituents in cereals can be acetylated at the *O*-2 position [6, 8, 9], and carry esterified ferulic acid (FA) or *p*-coumaric acid (*p*CA) linked at the *O*-5 position [7, 10]. Oxidative dimerization of feruloyl groups further increases the complexity of the xylan by forming intra- and inter polymeric cross-linked xylan chains, or interactions with lignins or proteins [10].

The degradation of xylan requires the cooperative action of different type of glycosyl hydrolases (GHs) and carbohydrate esterases (CEs), as classified in the CAZy database [11, 12]. GHs cleave the glycosidic bonds disrupting xylan backbone and removing arabinosyl or (4-*O*-methyl) D-glucuronyl decorations [12]. Carbohydrate esterases from the CAZy database families CE1-CE7 and CE16 remove acetyl esters from xylan while feruloyl esters are eliminated by enzymes presently only belonging to CE1 [13]. Microorganisms capable of fermenting arabinoxylan secrete GHs and CEs releasing mono- or oligosaccharides from the substrate, while some Gram-positive bacteria have further developed a more sophisticated system for depolymerizing part of the AX within their cytosol. To do so, they import extracellularly released xylo-oligosaccharides (XOS) and/or arabinoxylo-oligosaccharides (AXOS) and degrade them in the cytosol using a set of intracellular enzymes [14–22]. However, if the imported AXOS are acetylated or partially decorated by FA and *p*CA, their processivity within the cell is less well understood.

**Fig. 1:**
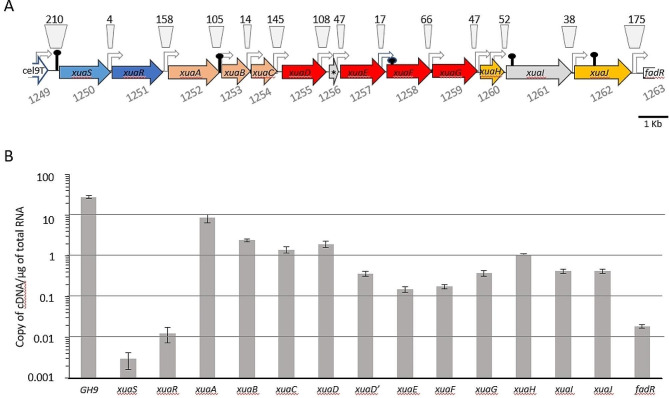
The *xua* genes and their expression. **A.** The *xua* gene cluster and surroundings genes displaying putative promoters (arrow) and terminators (stem-loop) predicted by BPROM and FindTerm softwares. The size of the intergenic regions in bp is indicated above the genes, and their locus number are indicated below. The genes in blue encode the sensor and the regulator forming a putative two-component system, genes in light brown, red, and orange designate the genes encoding the components of the ABC transporter, the GHs, and the esterases, respectively. Genes in grey correspond to genes encoding unknown function proteins. The star corresponds to gene *xuaD’*. **B.** Copy number of each gene per gram of mRNA extracted from *Ruminiclostridium cellulolyticum* grown in a minimal medium containing AX (WAXY-I)

We chose the model anaerobic Gram-positive bacterium *Ruminiclostridium cellulolyticum* to address this question. This versatile bacterium is capable to grow on AX, cellulose, xyloglucan and more complex natural substrates like wheat straw. On one hand, this organism produces extracellular multienzymatic complexes called cellulosomes for the degradation of plant cell wall polysaccharides; while, on the other hand, it has the capacity to import and intracellularly break down large AXOS of up to 6 monosaccharides through a system known as Xua (xylan utilization-associated genes) [14, 23–26]. The Xua system is encoded by a cluster of 13 genes (Fig. 1) and is important for the growth of the strain on arabinoxylan [14]. The genes *xuaA, B*, and *C* encode ABC-transporter components: XuaA, is a Solute Binding Protein binding to AXOS of up to 6 monosaccharides and XuaB and XuaC, form the membrane channel of the transporter. The downstream genes *xuaD*, *E, F*, and *G* encode intracellular GH51, 43, 8, and 39, respectively, that cleave arabinosyl decorations (XuaD and XuaE) and xylosyl units from the AXOS backbone (XuaF and XuaG). The genes *xuaH* and *xuaJ* encode two intracellular esterases, while *xuaI* encodes a protein of unknown function [14]. We observed that the genes *xuaABCD* are strongly up-regulated when *R. cellulolyticum* is cultivated on AX, but the genes downstream of *xuaD* do not show a similar trend [14]. Inactivation of *xuaA* by insertion of a type II intron caused a polar effect preventing expression of genes downstream of *xuaA* beyond *xuaD*. The trans-complementation with the genes *xuaABCD* failed to restore the wild-type growth phenotype on AX [14], indicating that despite the lack of up-regulation, the genes downstream of *xuaD* appear to play an important role in AX utilization.

XuaH and XuaJ previously showed acetyl esterase activity on 1-Naphthyl-acetate [14]. The Xua system seems therefore to include two acetyl esterases whose genes are poorly expressed when cells are cultured with arabinoxylan. In the light of these observations, we sought to establish whether these genes are indeed components of the *xua* system, and if so, whether XuaH and XuaJ exhibit different catalytic profiles. Our current research shows that the XuaH and XuaJ esterases target different acylations present in substrates. The *xuaABCDD’EFGHIJ* genes form a single operon indicating that the esterases do indeed belong to the Xua functional unit dedicated to AXOS degradation. Our study marks the first demonstration that cytosolic esterases are involved in the removal of at least feruloyl, and *p*-coumaroyl decorations from imported acylated AXOS.

## Material and methods

### Strains, media, vectors and primers

Strains, vectors and primers are reported in Table S1 and S2. *Escherichia coli* strains were grown at 37 °C in LB medium except specific mention, supplemented with appropriate antibiotics (100 µg. mL^− 1^ of ampicillin, 50 µg. mL^− 1^ of kanamycin, or 35 µg. mL^− 1^ chloramphenicol). *R. cellulolyticum* H10 ATCC 35,319 and mutants were grown anaerobically at 32 °C on basal medium [24] supplemented with either 2 g.L^− 1^ arabinose (Sigma-Aldrich, Darmstadt, Germany), or arabinoxylan from wheat flour (WAXY-I, Megazyme, Bray, Ireland).

### Construction of the *xuaS* and complemented mutant in *R. cellulolyticum*

The ClosTron method was used to construct mutants as previously described, using specific primers [27, 28]. The mutant strains interrupted in the gene located at the locus Ccel_1250 with group II intron was called MTL*xuaS*. Intron insertion was verified by PCR, and by Southern blot, as previously described [29].

PCR amplifying the genes *xuaSR* was performed from *R. cellulolyticum* genomic DNA using specific primers, amplicons, and pSOS956 vector digested with EheI and BamHI were ligated as formerly described [24], resulting in pSOS*xuaSR*.

### RNA preparation, reverse transcription

WT *R. cellulolyticum* and *xuaS* mutant derivative strains were grown in a minimal medium supplemented with AX (WAXY-I) until the mid- to the late-log exponential phase of growth, cells were harvested by centrifugation (7000 *g*, 5 min, 6 °C) then flash-frozen in liquid N_2_ before storage at -80 °C. Maxwell® 16 miRNA Tissue Kit (Promega, Madison, WI) was used for total RNA isolation. Extra DNase treatment and RNA quality control were performed as previously described [14].

### Quantitative real-time-PCR for transcriptional analyses

qPCR was performed on cDNA synthesized from mRNA as previously described [30]. For each point, technical duplicates and biological triplicates were performed. 16 S RNA encoding gene was used as the reference for normalization. Amplification efficiencies for each primer pair varied between 89.7 and 102.2%.

### Fluorescence measurements

Fluorescence measurements follow the same procedure as previously described [24]. The gene *xuaR* was amplified by PCR using the forward and the reverse primers *xuaR*1251pBadNheD and *xuaR*1251pBadSalR_NEW and the amplicon was digested by NheI and SalI before ligation with NheI-SalI linearized pBAD24 resulting in pBAD-*xuaR*. Intergenic regions (IR) located upstream of the genes *xuaS*, *A, D, D’, G, H*, and *I* were amplified using forward and reverse primers pairs and the amplicons were cloned in pUA66 by digestion with XhoI and BamHI, resulting in transcriptional fusions of IR with *gfpmut2* gene. MG1665 *E. coli* strain was then transformed with the either pBAD24 or pBAD24*xuaR* vector, and pUA66 derivatives. The fluorescence of transformants cultivated in minimal medium M9 containing 0.2% casamino acids (Gifco, USA), and appropriate antibiotics was measured [24].

### Protein production, SDS-PAGE and western blot analysis

Recombinant proteins expression and purification, cell extracts, SDS-PAGE and Western blot performed with rabbit primary antibody probing XuaA were performed as previously described [14, 31]. Briefly, *E. coli* BL21(DE3) was used as the recipient strain for the production of the recombinant proteins from the pET vectors (Table S1). After culture in LB and induction with IPTG, the cells were pelleted and broken in a French press. The cell extract was loaded on a Ni-nitriloacetic acid resin. Proteins eluted with imidazole were loaded on an ion-exchange chromatography column. Purified proteins were then concentrated and dialyzed in 25 mM potassium phosphate buffer (pH7) and their protein concentration was evaluated at 280 nm using their respective extinction coefficient.

### Enzyme activity measurement

Esterase activity was tested using methyl-ferulate, methyl-*p*-coumarate, methyl-sinapate, and methyl-caffeate. Hydrolysis of methyl-esters (50 µM) in MOPS 100mM pH6) was evaluated by measuring the change of the spectra during 1 to 10 min at 335 nm. Enzymes were tested at a concentration of 1 µM for screening purpose, then a concentration of 10 nM was used for specific activity measurement.

Specific activities were also determined using *p*-nitrophenyl acetate (*p*-NPA), *p*-nitrophenyl butyrate (*p*-NPB), and *p*-nitrophenyl trans-ferulate (*p*-NPFA) (Sigma-Aldrich). The release of *p*-nitrophenol (*p*-NP) was monitored at 400 nm for 1 min at 37 °C in 50 mM phosphate buffer pH 7, with a substrate final concentration of 0.1mM for *p*-NPFA and *p*-NPA, and 1mM *p*-NPB. Stock solutions of *p*-NPFA and *p*-NPB were dissolved in DMSO, in this case, the reaction buffer was supplemented with 1% v/v Tween 80. Finally, enzyme concentration for XuaH and XuaJ was set at 1 µM.

### Activity on raw substrates

Hatched wheat bran (Markal, Saint Marcel-les-Valence France) and wheat straw (Valagro, Poitier, France) substrates were rinced with water and dried. These materials (20 g. L^− 1^) were incubated in a final reaction volume of 1 mL in MOPS 100 mM pH 6.0 with a mixture of 1 µM *Neocallimastix patriciarum* xylanase (Megazyme, E-XYLNP) and 1 µM of each *R. cellulolyticum* α-arabinofuranosidases XuaD and XuaE [14]. Reactions were performed with or without XuaH (0.8 µM) or XuaJ (0.8 µM). The mixture of all enzymes was incubated for 2 h at 37 °C under shaking at 800 rpm, and 2 h at 50 °C (800 rpm). Then additional XuaH (0.8 µM) or XuaJ (0.8 µM) was added (or not) and incubated for another 2 h (37 °C, 800 rpm) in the respective reaction mix. The samples were then boiled (5 min), centrifuged (20,000 *g*, 5 min) and the supernatant was stored at -20 °C before analysis.

To analyze their content in free hydroxycinnamic acids (ferulic and coumaroyl acid), the samples were filtered (0.45 μm PVDF syringe filter) prior to HPLC analysis at 220 nm and 40 °C on a model Agilent1100 (Agilent Technologies, Massy, France), equipped with a variable UV/Vis detector and a 100-position autosampler autoinjector. Separations were achieved on a C30 reversed-phase column (YMC™ Carotenoid 3 μm, 4.6 × 150 mm, Waters, Guyancourt, France) at 0.8 mL/min, with solvent A: water acidified by 0.05% phosphoric acid and acetonitrile (95:5, v/v), and solvent B: acetonitrile 100%. The gradient program was: solvent B 10% for 5 min, 10 to 35% in 15 min, and 35 to 90% in 5 min. It remained at 90% for 3 min to finally reach 100% in 1 min. The Agilent1100 ChemStation processed the data, and the quantification was performed by external standard calibrations.

### Bioinformatic analysis

DNA sequences were analyzed using BPROM and FindTerm online softwares [32]. For protein sequences, the analysis was performed using the ESTHER database (http://bioweb.ensam.inra.fr/esther) [33], the Carbohydrate Active Enzymes database (http://www.cazy.org/) [11], or the Protein BLAST search at NCBI (https://blast.ncbi.nlm.nih.gov/Blast.cgi) and ClustalW [34].

### Model building and docking experiments

Model building was performed using the whole sequence of XuaH of 265 residues on the free COLAB version of Alphafold variant ColabFold which uses the MMesqs2 server to calculate the multiple sequence alignment [35, 36]. The best of the 5 calculated models had a very high confidence index (lDDT) above 90 except for a hydrophilic and maybe disordered loop region (res25-45) located on the back side of the enzyme. Docking was performed with the SMINA variant of the Autodock/Vina program under windows, using an in-house created plugin (GitHub) with the PyMol structure visualization program [37]. All ligands were constructed based on monomer fragments from the PDB databank, assembled and their energy minimized with the free AVOGADRO program [38].

## Results

### XuaR regulates *xuaA* gene expression

We previously reported the presence of transcriptional intergenic links between two- and/or three- successive genes of the cluster from *cel9T* to *xuaJ* suggesting an operon structure covering all *xua* genes [14]. To go further, we analyzed the expression profile of *xua* and surrounding genes under induction conditions (AX). The level of the transcripts from *xuaA* to *xuaJ* is higher than those of *xuaSR* and *fadR*, which is consistent with an operonic structure covering the genes *xuaA* to *xuaJ* (Fig. 1B).

**Fig. 2:**
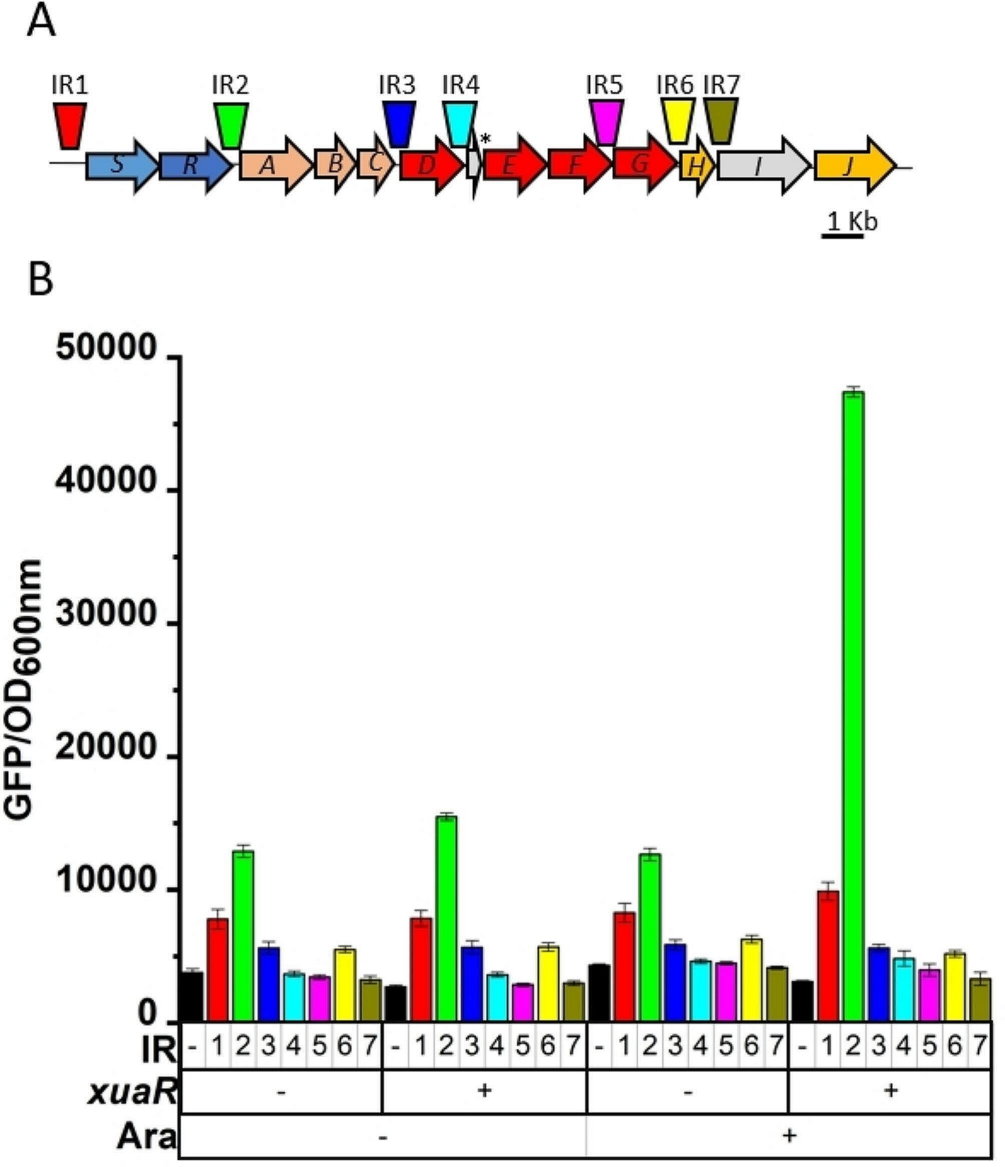
Targets of the response regulator XuaR. (**A**) Localization and size of the intergenic regions tested in the *xua* cluster from IR1 to IR7. Letters in the genes correspond to the last letter of each corresponding *xua* gene. (**B**) Fluorescence intensity of recombinant *E. coli* MG1655 containing pBAD24 or pBAD*xuaR* (*xuaR -* or *xuaR* +) and different derivatives of the pUA66 carrying intergenic regions according to their color code in A, or not (black). Arabinose was added or not to the medium (Ara + or Ara -). Arabinose allows induction of the expression of *xuaR* in the pBAD*xuaR* vector. Experiments were performed in triplicate and bars indicate the standard deviation

Upstream of *xuaA*, the genes *xuaS* and *xuaR* encode proteins predicted to form a two-component system, XuaS being a sensor histidine kinase of the LytS family, and XuaR acting as a two-component regulator of the AraC family. To test whether XuaR targets the expression of genes of this operon, we reconstituted the regulatory pathway in *E. coli* as described previously [24]. Intergenic regions (IR) located upstream of *xuaA*, and other genes were tested (Fig. 2). Of these, only IR2, located upstream of *xuaA* was able to induce the expression of the reporter gene in *E. coli*, showing that XuaR might therefore control the expression of *xuaA* and the downstream genes of the operon in *R. cellulolyticum*.

### XuaS mutant growth is impaired on arabinoxylan

Introduction of a vector allowing the expression of *xuaABCD* genes in the previously constructed *xuaA* mutant failed to restore a normal growth on AX, suggesting that the genes located downstream of *xuaD* are important for growth on this decorated xylan [14]. To assess this hypothesis, we analyzed the growth of three newly constructed strains on AX: (i) the *xuaS* mutant strain in which *xuaS* was inactivated by insertion of a type II intron (*MTLxuaS*) resulting in a non-functional two-component system (Figure S1), and the *xuaS* mutant strain transformed either (ii) with a vector containing the genes *xuaSR* (pSOS*xuaSR*) or (iii) with an empty vector (pSOSzeroTm).

**Fig. 3:**
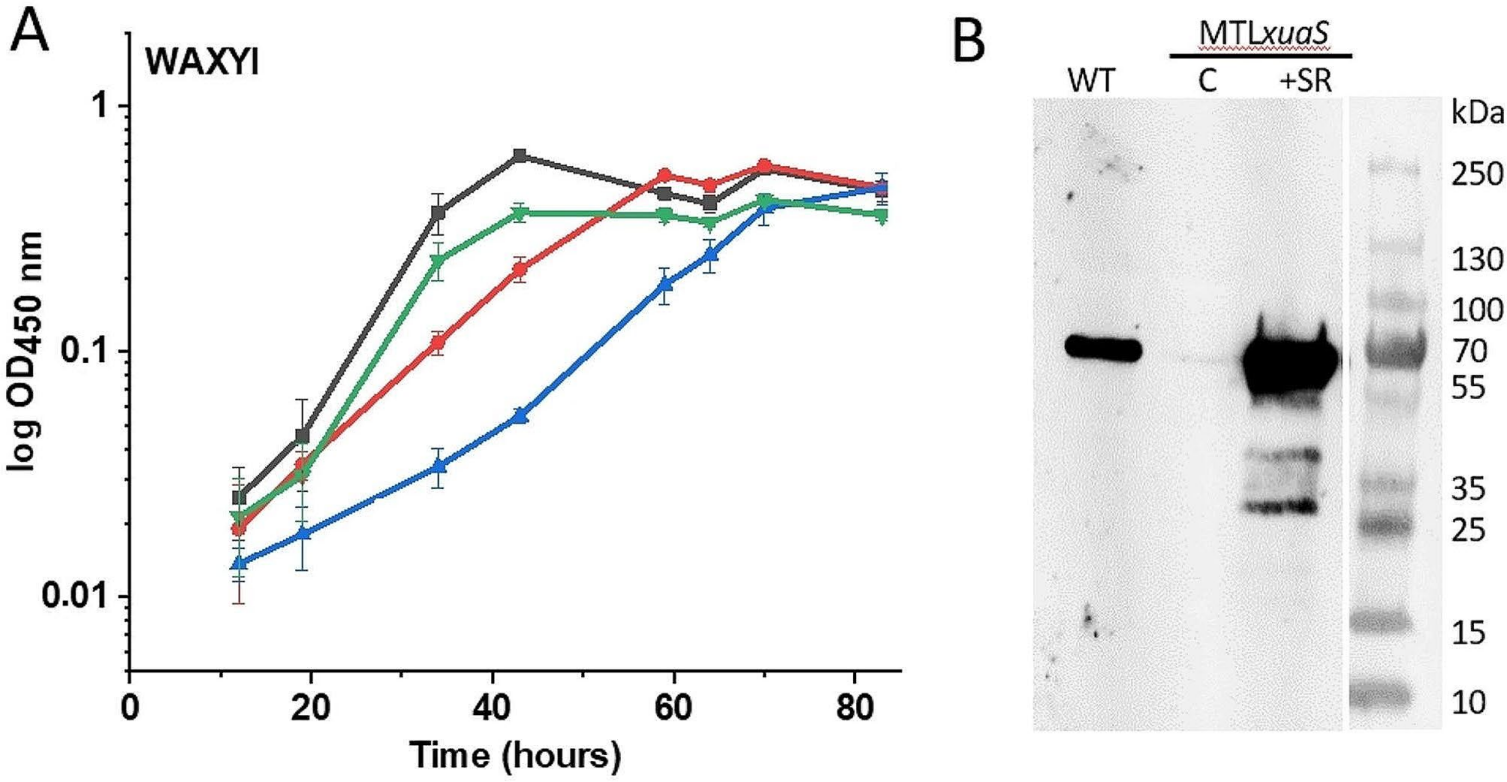
Characterization of wild-type and MTL *xuaS* derivative strains. (**A**) Growth curve of the different strains grown in minimal medium containing 2 g L^− 1^ AX. In black, WT strain (5.6 ± 0.4 h doubling time), in red, MTL*xuaS* strain (9.5 ± 0.4 h doubling time), in blue, MTL*xuaS* strain carrying an empty vector (9.8 ± 0.6 h doubling time), in green, MTL*xuaS* strain carrying pSOS*xuaSR*, (6.3 ± 0.6 h doubling time). Experiments were performed in triplicate and bars indicate the standard deviations. (**B**) Western blot analysis using anti-XuaA antiserum. An equivalent amount of proteins of cell extract was loaded from *R. cellulolyticum* wild-type (WT), MTL*xuaS* containing the vectors pSOSzeroTm (**C**), or pSOS*xuaSR* (+ SR)

The *xuaS* mutant and its derivative strains carrying either the pSOS*xuaSR* or the control empty vector pSOSzeroTm grew similarly to the wild-type strain in a minimal medium containing arabinose as the sole carbon source, indicating that the inactivation of *xuaS* and the presence of these vectors do not affect the fitness of the strains (Figure S2). When using AX as the sole carbon source, growth of the *xuaS* mutant is characterized by an almost 1.7-fold increase of the doubling time compared with the wild-type strain (Fig. 3A). This phenotype is consistent with the phenotype previously observed for the *xuaA* mutant strain [14]. The strain MTL*xuaS* (pSOS*xuaSR*) grows similarly to the wild-type strain thereby indicating the successful restoration of the wild-type phenotype of the MTL*xuaS* strain on AX (Fig. 3A). Using an antiserum raised against XuaA, western blot analyses confirmed that both the wild-type and the MTL*xuaS*(pSOS*xuaSR*) mutant strain produce XuaA, in contrast to the MTL*xuaS* mutant strain containing the pSOSzeroTm control vector (Fig. 3 and Figure S3). This result confirmed that the phenotype of the MTL*xuaS* strain observed when grown on AX is at least linked to the drastic reduction of the production of XuaA. It also confirms that the XuaSR two-component system controls *xuaA* expression and that it is important for growth on AX. The transformation of the MTL*xuaS* mutant strain with the pSOS*xuaSR* vector restores the wild-type phenotype of the mutant strain. This is probably due to the induced expression of the entire operon, including the genes located downstream of *xuaD*.

### *xuaH, I, J* genes are overexpressed in the complemented *xuaS* mutant strain

**Fig. 4:**
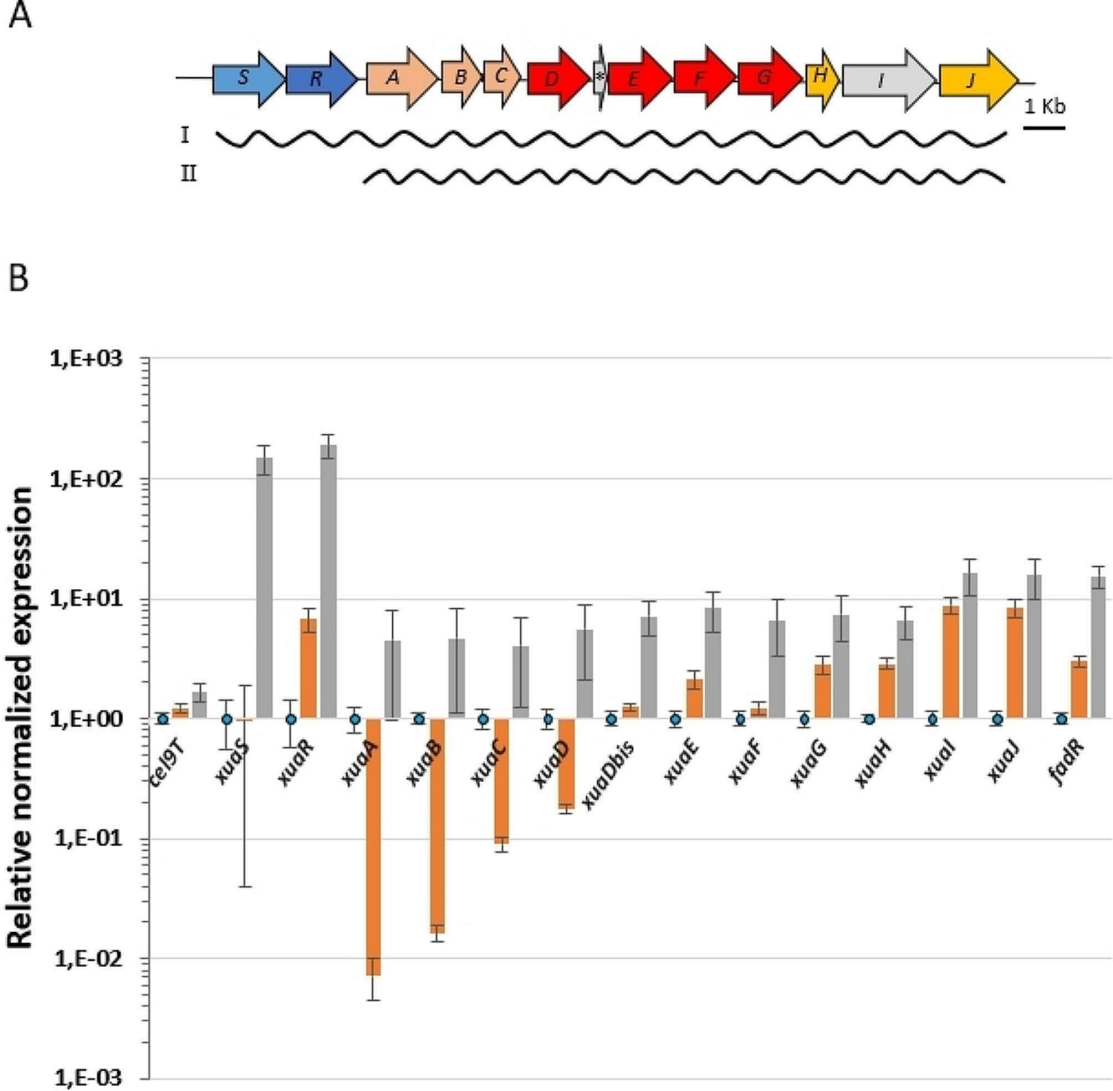
qPCR analysis of mRNA produced by wild-type and derivative strains. (**A**) The *xua* cluster and its putative transcripts I and II. (**B**) qPCR, total RNA was extracted from cultures of *R. cellulolyticum* wild-type (blue bars), *MTLxuaS* mutant strain containing either the pSOSzeroTm (orange bars) vector or the pSOS*xuaSR* (grey bars), grown in minimal medium supplemented with 2 g L^− 1^ AX (arabinoxylan), as the sole carbon source. Normalization was performed using the 16 S RNA encoding gene and gene expression level is given relative to wild-type strain culture. Error bars indicate the standard deviations of three independent experiments

The expression level of the *xua* genes in the *xuaS* mutant strain containing the pSOS*xuaSR* or pSOSzeroTm vectors was compared with that of the wild-type strain, under inducing condition, i.e. in the presence of AX-based culture medium (Fig. 4). As expected, *xuaS* and *xuaR* are highly expressed from the pSOS*xuaSR* vector in the strain MTL*xuaS* (pSOS*xuaSR*). In this strain and under inducing conditions, XuaSR presence results in a four- to sixteen-fold increase of the expression level of *xua* genes compared with the wild-type strain, including the more distant *xuaH, xuaI,* and *xuaJ* (Fig. 4). This result confirmed that these genes are all part of the same operon regulated by XuaR.

In the MTL*xuaS*(pSOSzeroTm) strain, *xuaABCD* genes are clearly down-regulated compared with the wild-type strain, but, the level of expression of *xuaG*, *xuaH*, *xuaI*, and *xuaJ* genes is increased (Fig. 4). This suggests a second level of control of the expression of the latter genes independent of XuaSR, and the probable presence of internal promoter(s) that might be activated by another inducing system. In conclusion, the *xua* genes form two operons, one which might cover the *xuaSRABCDD’EFGHIJ* genes as deduced from intergenic transcriptional links experiments [14], and a second one covering the *xuaABCDD’EFGHIJ* genes, whose expression is induced by XuaSR in the presence of AX in the culture medium (Fig. 4).

### Activity of XuaH and XuaJ on synthetic and natural wheat substrates

XuaH and XuaJ were both found to have acetyl esterase activity on the synthetic substrate, 1-Naphtyl acetate [14]. However, during growth on natural substrates, AXOS generated from AX are likely to carry feruloyl or *p*-coumaroyl decorations, as commonly found in cereal AX. This prompted us to further study the activity of XuaH and XuaJ, and explore their ability to remove phenolic decorations present on Ara*f*.

The esterase specificity and activity of XuaH and XuaJ was first tested on *p*NP-acyl esters. XuaH is more active on *p*NP-butyrate, followed by *p*NP-ferulate and *p*NP-acetate (Table 1) whereas XuaJ is strongly active on *p*NP-acetate, but is only weakly active on *p*NP-butyrate and is not active on *p*NP-ferulate. The specificity of XuaH and XuaJ esterases was then examined with the hydroxycinnamate methyl-esters: methyl ferulate (MFA), methyl *p*-coumarate (M*p*CA), methyl sinapate (MSA) or methyl caffeate (MCA). Unlike XuaJ, XuaH is able to cleave all the ester bonds of all four of these methyl esters aromatic derivatives. The determined specific activity for each substrate in decreasing order was: MFA (2028 ± 141 IU/µmol) > MSA (607 ± 32 IU/µmol) > MpCA (305 ± 32 IU/µmol) > MCA (118 ± 13 IU/µmol). XuaH is therefore versatile in terms of substrate specificity.

**Table 1:** Specific activity measured with *p*NP-acyl esters

	*p*-NP-ferulate	*p*-NP-acetate	*p*-NP-butyrate
XuaH	4683 ± 218	1555 ± 11	6941 ± 182
XuaJ	NA	18,668 ± 700	125 ± 3

Activity is given in IU/µmol. NA, no activity

**Fig. 5:**
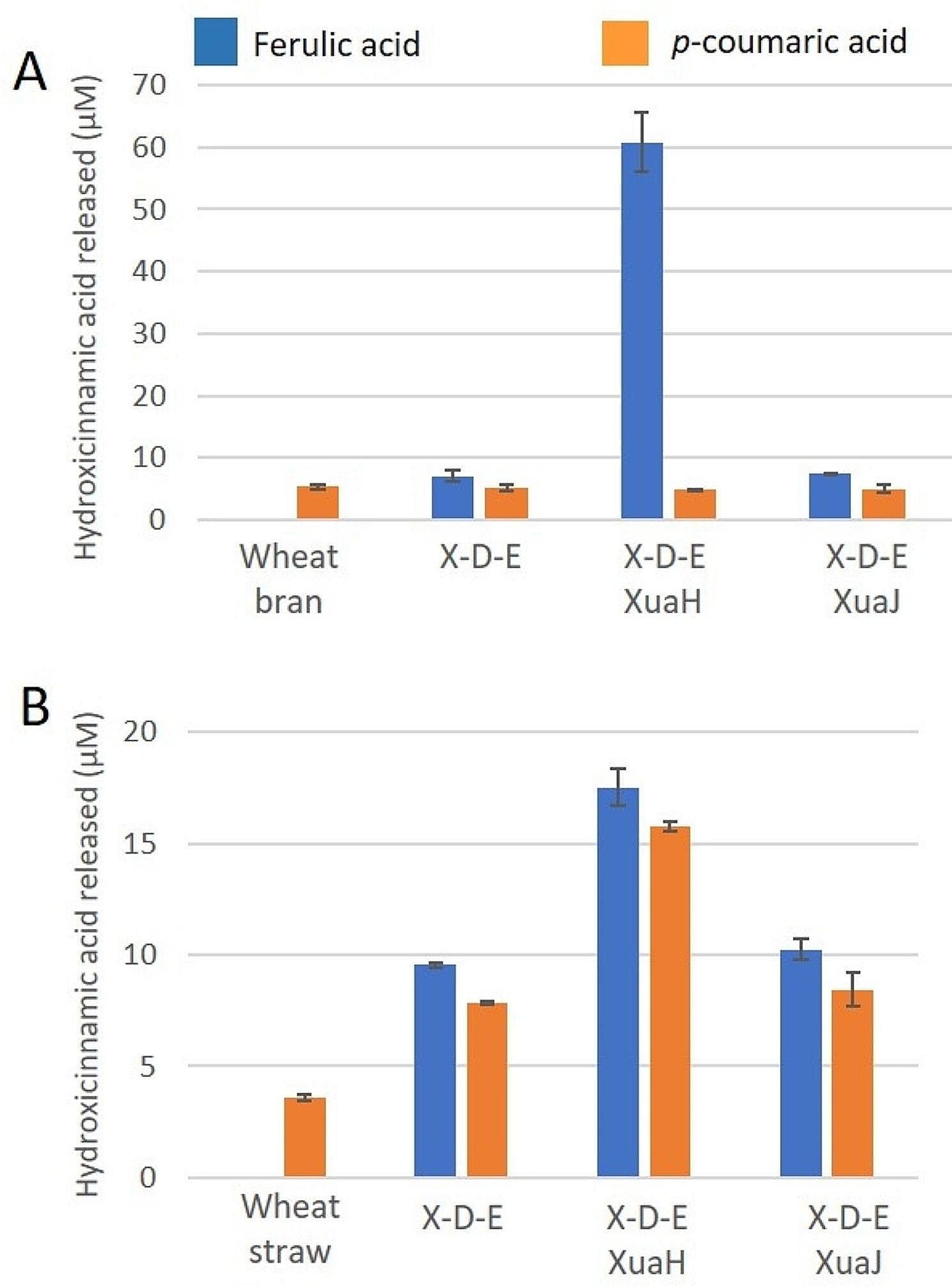
Activity of XuaH and J on natural wheat-derived substrates. HPLC analysis of the content of ferulic acid (blue) and *p*-coumaric acid (orange) released from wheat bran (**A**) or wheat straw (**B**) by XuaH or XuaJ. Wheat bran and wheat straw were pre-treated with a mixture of commercial xylanase with the α-arabinofuranosidases XuaD and XuaE (X-D-E)

The activity of XuaH and XuaJ were then tested on a more complex natural substrate. They were incubated with wheat bran or wheat straw since arabinoxylan is their predominant hemicellulosic polysaccharide [39]. No change in peaks corresponding to ferulic or *p*-coumaric acids were detected in the HPLC analysis of the substrate incubated with any of these enzymes. As XuaH and XuaJ are intracellular enzymes, we hypothesized that they may be active on oligosaccharides (imported by the ABC-transporter) rather than on polysaccharide. Thus wheat bran and wheat straw were preincubated with a mixture containing a commercial *endo*-xylanase from *Neocallimastix patriciarum* and the two Xua α-L-arabinofuranosidases from *R. cellulolyticum*, previously characterized (XuaD and XuaE) [14] to facilitate the accessibility of the xylanase to the substrate. HPLC analysis showed that FA was released only by XuaH with a level corresponding to 30% of the total alkali-extractable FA (206 µM FA) quantified from wheat bran (Fig. 5 and Figure S4)). No *p*CA was released by both enzymes, which is in line with the analysis of wheat bran composition showing levels below the level of detection of this compound. On wheat straw that contains both FA and *p*CA (192µM FA and 452 µM pCA alkali extractable), twice the levels of pCA and FA were released in the presence of XuaH compared to its absence (Fig. 5 and Figure S4).

### Modeling of XuaH

**Fig. 6:**
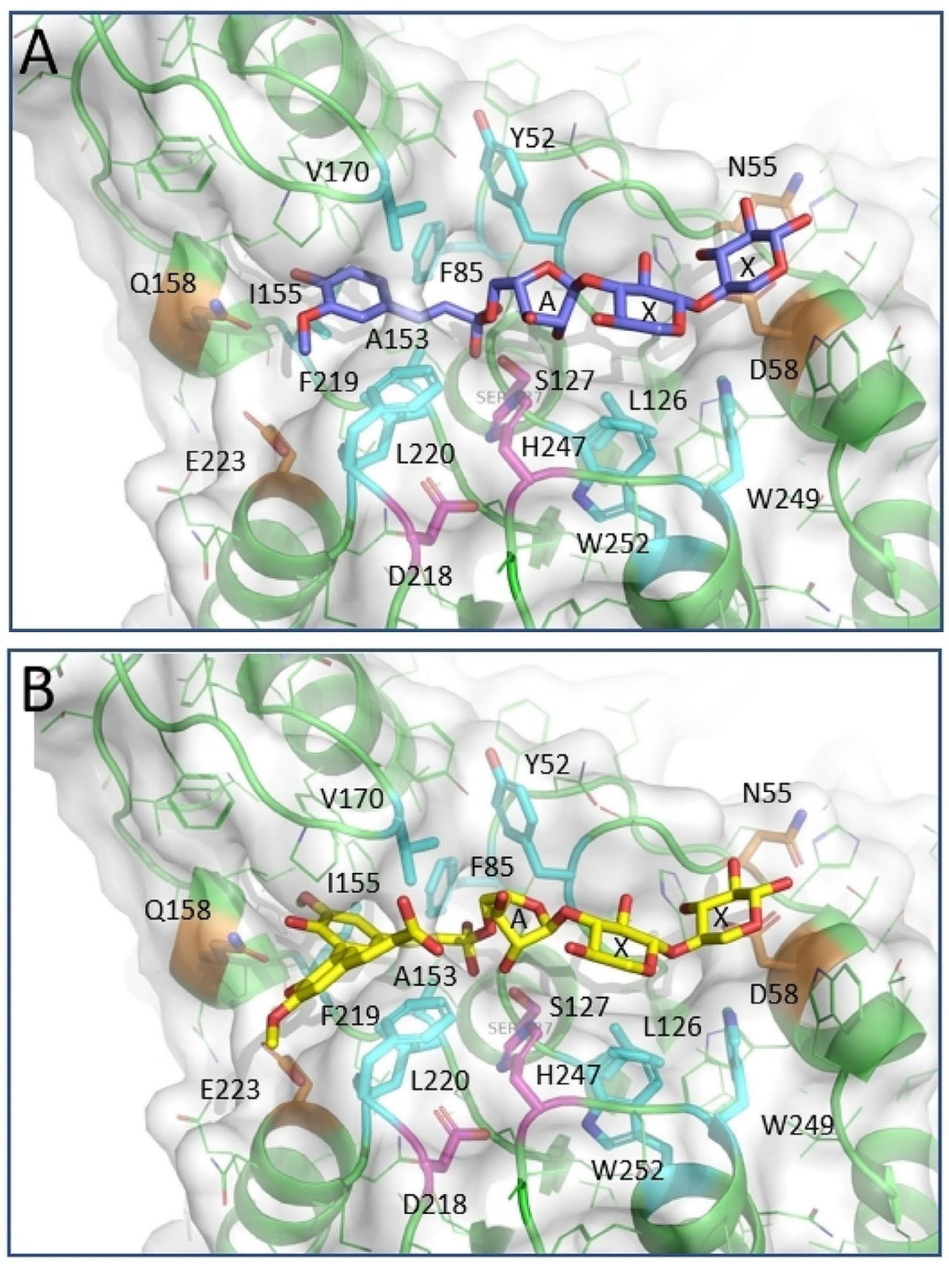
Model of XuaH. Views of the best fit of the transition state of feruloyl-arabinoxylobiose (in blue) (**A**) and the transition state of diferuloyl-arabinoxylobiose (in yellow) (**B**), docked in the active site of the ALPHAFOLD XuaH model as obtained with AUTODOCK/SMINA. The docking was performed with the Ser127Gly mutant of XuaH to prevent steric hindrance of the transition state. The catalytic triad including the omitted nucleophile Ser127 is highlighted in magenta. The hydrophobic and hydrophilic amino acids cited in the text are highlighted in cyan and in orange, respectively. Both transition states fit in the active site and are stabilized. A mean arabinofuranose, X means xylose

A model of XuaH was constructed with the COLAB version of Alphafold (Figure S5). The obtained result had a high reliability index (pLDDT) of 95.7 with a ptm score of 93.4. Its fold is an α/β- hydrolase fold typical for esterases and contains a catalytic triad composed of Ser127, His247, and Asp218. The triad is located on the top of a parallel β-sheet composed of 6 strands, with the nucleophile Ser127 located in the center capping the N-terminal side of a helix pointing towards the active site. This arrangement creates a positively charged environment favorable for the stabilization of the negatively charged oxyanion intermediate of the reaction. The lower section of the active site pocket is formed of mostly hydrophobic and aromatic residues like Leu126, Trp252, Trp249, Phe219, Leu220, Ala 153, Ile 155, Val170, Phe85, Tyr52 with hydrophilic residues flanking both extremities Glu223 and Gln158 on one side and Asp 58 and Asn55 on the other (Fig. 6). The pocket is accessible from the solvent and large enough to host an oligosaccharide. We performed molecular docking with Smina to probe the accessibility of the side for various xylan degradation products of different lengths [37]. Among the tested dextrins (mono- or diferuloyl-arabinoxylose to arabinoxylotriose), it appeared that the pocket may best stabilize an oxyanion intermediate of a feruloyl-arabinoxylobiose (feruloyl-5-*O*- L-arabinofuranosyl α-1,3 D-xylosyl β-1,4 D-xylose) or diferuloyl-arabinoxylobiose (di-dehydroferuloyl-5-*O*- L-arabinofuranosyl α-1,3 D-xylosyl β-1,4 D-xylose) oxyanion (Fig. 6).

## Discussion

Our study shows that the *xuaH*, *I* and *J* genes are part of the *xuaABCDD’EFGHIJ* operon, which is regulated by the XuaSR two-component system. The operon forms a functional unit responsible for importing and depolymerizing AXOS in the cytosol. The signal detected by XuaS is unknown, but it is not arabinose or xylose, as their presence in the culture medium does not induce expression of the *xua* operon [14]. Our genetic approach has revealed that the expression of genes located at the end of the cluster (*xuaGHIJ*) may also be under the control of additional regulatory mechanism(s). Their expression could be activated as needed, independent of XuaSR. It is possible that XuaH and XuaJ are required to remove acyl groups from xylodextrins and/or feruloylated GAX-dextrins, which may be imported by other ABC transporter(s).

The before-mentioned results indicate that XuaJ and XuaH can remove acetyl decorations from synthetic substrates but their activity could not be verified on natural substrate in our experiments. XuaH is found to cleave feruloyl and *p*-coumaroyl decorations from both synthetic and natural substrates, and according to the model, could also release diferulic acid. In the ESTHER database [33], XuaH is annotated as a putative esterase in NCBI and belongs to the A85-esteraseD-FGH family. This family is a subset of the CE1 family which is the only family associated with feruloyl esterase activity. A BLAST search using the primary sequence XuaH with the Refseq_select_prot database in NCBI, yielded proteins with identity scores ranging between 50 and 90%. The proteins with the highest score are found in bacteria phylogenetically related to *R. cellulolyticum* like *Ruminiclostridium josui* (89% identity), *Ruminiclostridium papyrosolvens* (88% identity), *Ruminiclostridium cellobioparum* (69% identity) suggesting that these bacteria share the same ability to cleave feruloyl esters in the cytosol. A BLAST search in the non-redundant Uniprot/Swissprot database yielded a single result, namely the acetyl esterase XynC from *Caldocellum saccharolyticum* (*Caldicellosiruptor saccharolyticus*) (55% identity). This enzyme is active on α-naphthyl-acetate but its activity was unfortunately not tested with hydroxycinnamate esters [40]. Interestingly XynC also lacks a secretory signal peptide, suggesting an intracellular localization as for XuaH. To our knowledge, a few bacterial feruloyl esterases lacking a signal peptide have been characterized to date. CaeA encoded in a locus for plant polysaccharides utilization in *Bifidobacterium longum* is active on MpCA, MCA and MFA but not MSA [41], FaeII and FaeIII from *Cellulosilyticum ruminicola* H1 are active on the four hydroxycinnamic methyl esters [42]. Tx-Est1 from *Thermobacillus xylanolyticus* and RuFae2 originating from a rumen microbial metagenome are active on MFA, MSA, M*p*CA and release ferulic acid from natural arabinoxylan [43, 44]. In addition, RuFae2 is active on MCA, Tx-Est is active on pNP-acetate and releases *p*-coumaric acid. Only Tx-Est releases diferulic acid from natural substrates. Like XuaH, these intracellular feruloyl esterase have rather broad substrate specificity. Similar to XuaH in *R. cellulolyticum*, XynC, FaeII and FaeIII, RuFae2, and Tx-Est1 might be involved in a system of import and intracellular degradation of xylan degradation products.

XuaJ does not display any similarity with proteins in the ESTHER database but belong to the newly created CE20 family of the CAZy database and is the second characterized member of this newly created family. XuaJ shares 37% identity with a xyloglucan acetyl esterase XacXaeA (XAC1771) from *Xanthomonas citris* pv. citri str. 306, which is the only enzyme characterized so far in the CE20 family [45]. XuaJ shares the same general domain organization, comprising two sialate *O*-acetylesterase domains (SASA) separated by a glycan-binding module. Primary sequence alignment suggests that the catalytic triad is formed by S104, D508, and H510 in XuaJ, which corresponds to the catalytic triad S115, D510, and H512 in XacXaeA (Figure S6). In this new CE20 family, XacXaeA and XuaJ have little or no activity on substrates longer than acetate, respectively. XacXaeA targets acetylated xyloglucan, while XuaJ could target acetylated arabinoxylan. Even if this activity was not verified on natural carbohydrate substrates, this enzyme might represent a new specificity in this family.

The model bacterium *R. cellulolyticum* has developed both extra- and intra-cellular strategies for breaking down AX. Its extracellular predicted or characterized cellulosomal endoxylanases, arabinofuranosidases, acetylesterases, and feruloylesterases, can first disconnect arabinoxylan chains that are linked together or to lignin in natural substrates, releasing dextrins [23, 25, 46]. The Xua system may import and break down AXOS with a wider range of diversity than expected, including AXOS with one or two arabinosyl, as well as acetyl, diverse hydoxycinnamic groups (feruloyl, *p*-coumaroyl), and possibly diferuloyl groups. Most Gram-positive bacteria with systems for importing and intracellular degrading of extracellular xylan degradation products have predicted or characterized acetylesterases but no feruloyl or cinamoyl esterase activity were reported [15–18, 20, 21, 47]. Our study on *R. cellulolyticum* demonstrates that this model bacterium possesses the enzymatic equipment to remove diverse hydroxycinnamic groups from the AXOS and to potentially cleave their acetyl decoration to allow their use in the catabolism. This remarkable versatility enables the bacteria to utilize many AXOS released from natural arabinoxylan raw substrates, giving it a competitive advantage for growth.

## Conclusion

The genes *xua*, including the *xuaH*, *I* and *J*, are part of the same operon. This operon is regulated by the two-component system XuaSR, forming a functional unit. The products XuaJ and XuaH thus belong to the system dedicated to the import and full depolymerization of AXOS in the cytosol.

XuaH is a versatile esterase that can hydrolyse feruloyl, *p*-coumaryl and diferuloyl esters, while XuaJ is an acetyl esterase. These cytosolic esterases enable *R. cellulolyticum* and other similarly equipped species to exploit AXOS with various esters released from crude arabinoxylan substrates, which can be imported into the cell. This reduces the need for extensive extracellular esterase processing and gives bacteria a growth advantage in a competitive environment. This complete system of regulation, uptake and intracellular degradation of acylated AXOS could serve as a template for developing engineered strains to produce biofuels or chemicals from lignocellulosic waste.

### Electronic supplementary material

Below is the link to the electronic supplementary material.

**Additional file 1: Figure S1.** Molecular analysis of the *Ruminiclostridium cellulolyticum* mutant strain. **Figure S2.** Growth of wild-type and *Ruminiclostridium cellulolyticum* mutant strains in arabinose-containing medium. **Figure S3.** Raw data of the Western blot analysis. **Figure S4.** Chromatograms of the HPLC analysis. **Figure S5.** Overall view of the fold of the alphafold model of XuaH. **Figure S6.** Alignment of XuaJ with XacXaeA. **Table S1.** Table of strains and vectors used in the study. **Table S2.** Table of Primers used in the study.

## Data Availability

No datasets were generated or analysed during the current study.

## Abbreviations

ABC: ATPase Binding Cassette
AX: Arabinoxylan, Xua, xylan utilization-associated genes
AXOS: Arabinoxylo-oligosaccharide, GAX, glucuronoarabinoxylan
GX: Glucuronoxylan, CE, Carbohydrate esterase, GH, glycosyl hydrolase
MFA: Methyl ferulate
M*p*CA: Methyl *p*-coumarate
MSA: Methyl sinapate
MCA: Methyl caffeate
