# Supplementary material for: Intracellular removal of acetyl, feruloyl and p-coumaroyl decorations on arabinoxylo-oligosaccharides imported from lignocellulosic biomass degradation by Ruminiclostridium cellulolyticum
Source: Microb Cell Fact. 2024 May 24;23:151. doi: 10.1186/s12934-024-02423-z (PMC11127375; doi:10.1186/s12934-024-02423-z)
Supplement: Supplementary file 1 — Additional file 1: Figure S1. Molecular analysis of the Ruminiclostridium cellulolyticum mutant strain. Figure S2. Growth of wild-type and Ruminiclostridium cellulolyticum mutant strains in arabinose-containing medium. Figure S3. Raw data of the Western blot analysis. Figure S4. Chromatograms of the HPLC analysis. Figure S5. Overall view of the fold of the alphafold model of XuaH. Figure S6. Alignment of XuaJ with XacXaeA. Table S1. Table of strains and vectors used in the study. Table S2. Table of Primers used in the study. [file 12934_2024_2423_MOESM1_ESM.pdf]

Figure S1. Molecular analysis of the *Ruminiclostridium cellulolyticum* mutant strain

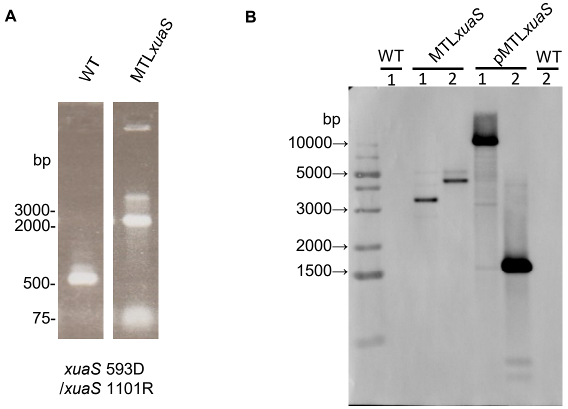

A. PCR analysis of WT gDNA and MTL*xuaS* strain cells using the primer pair *xuaS* 593D / *xuaS* 1101R hybridizing upstream and downstream of the targeted type II intron insertion site in *xuaS*. The expected size of the amplicons is 528 bp in WT and 2309 bp when type II intron is inserted in *xuaS* in the *MTLxuaS* mutant strain.

B. Southern blot. WT gDNA, MTL*xuaS* mutant gDNA, and pMTL*xuaS* plasmid were cut with HindIII (1) or HaeIII (2). After migration and transfer, the membrane was incubated with a labeled probe targeting the erythromycin resistance cassette. The size of the detected fragments in MTL*xuaS* is consistent with the theoretical one (3336 bp for HindIII and 4386 bp for HaeIII). Only one insertion is detected.

Figure S2. Growth of wild-type and *Ruminiclostridium cellulolyticum* *xuaS* mutant strains on arabinose-containing medium

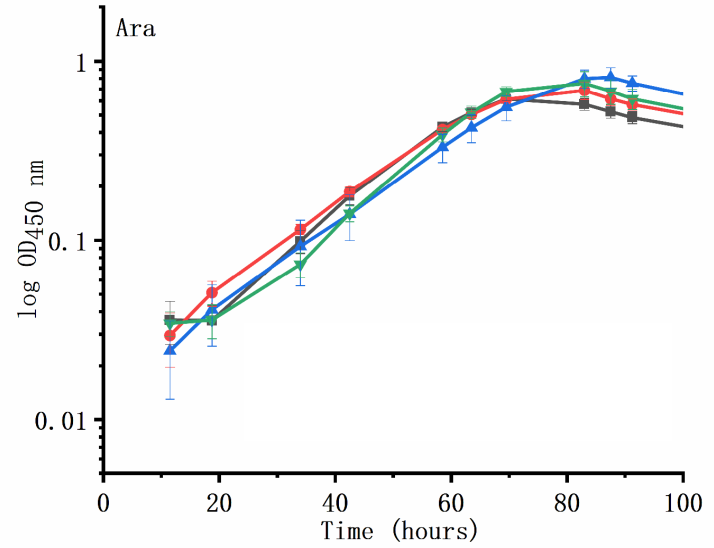

The strains were grown on a minimal medium containing 2 g L^{-1} arabinose. The strains are WT (gray), mutant strains MTL*xuaS* (red), MTL*xuaS* strain carrying an empty vector pSOSzeroTm (blue), and pSOS*xuaSR* (green). Experiments were performed in triplicates and bars indicate the standard deviation.

Figure S3. Raw data of the Western blot analysis

A

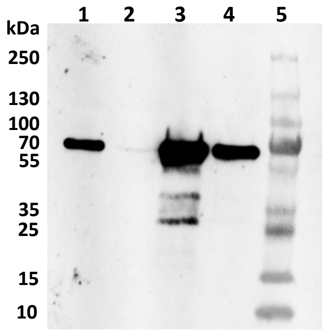

1. WT
2. MTL*xuaS*_pSOSzeroTm-Cl.A
3. MTL*xuaS*_pSOS*xuaSR*
4. MTL*xuaS*_pSOS*xuaSR*
5. Ladder

A. Western blot using anti-XuaA serum

B. Membrane of the Western blot stained with Ponceau S to reveal the blotted proteins before probing with the antibody

C. SDS-PAGE of the same samples stained by Coomassie Blue

B

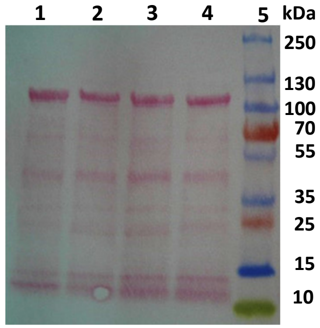

C

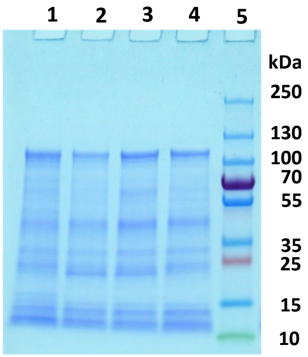

Figure S4. Chromatograms of HPLC analysis

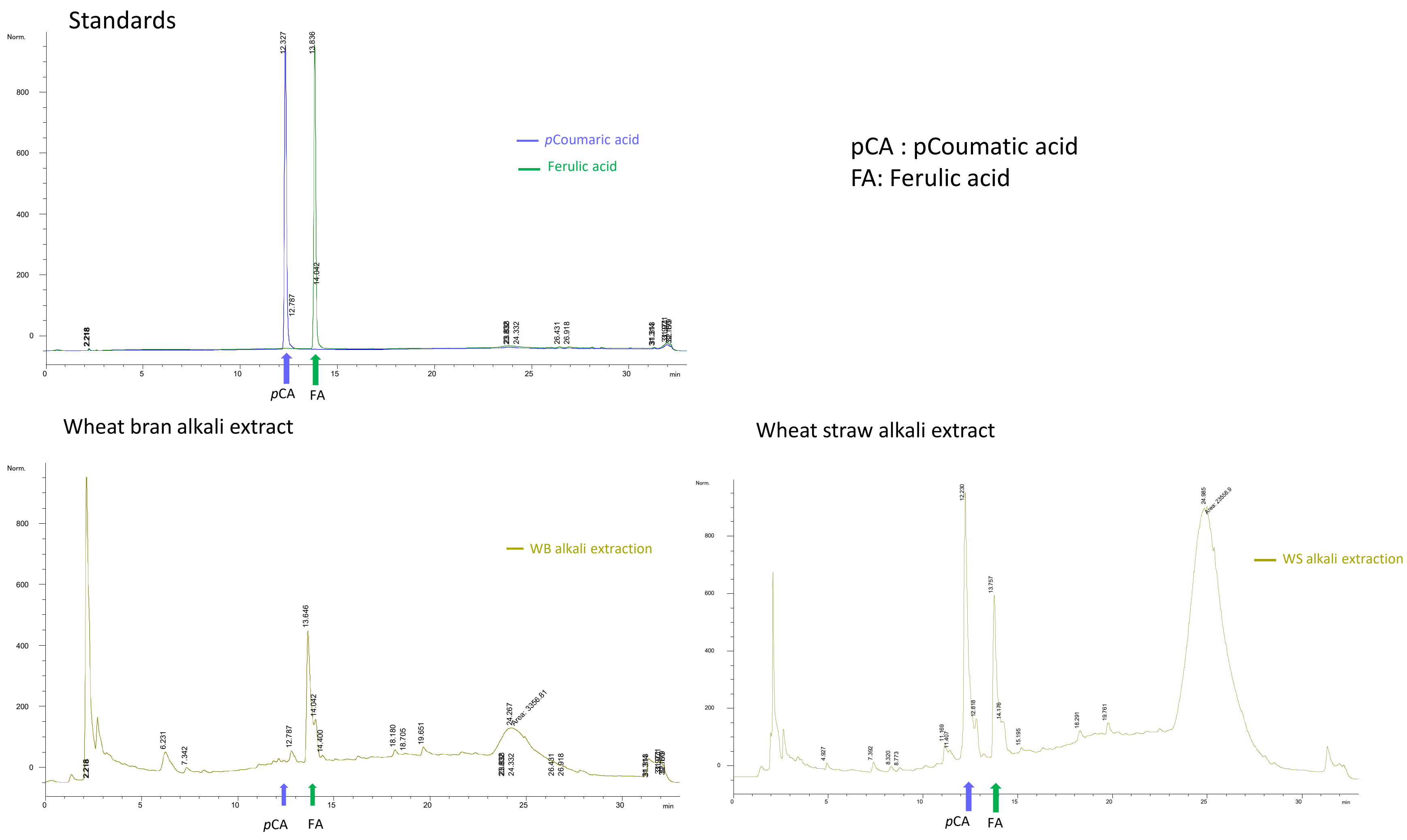

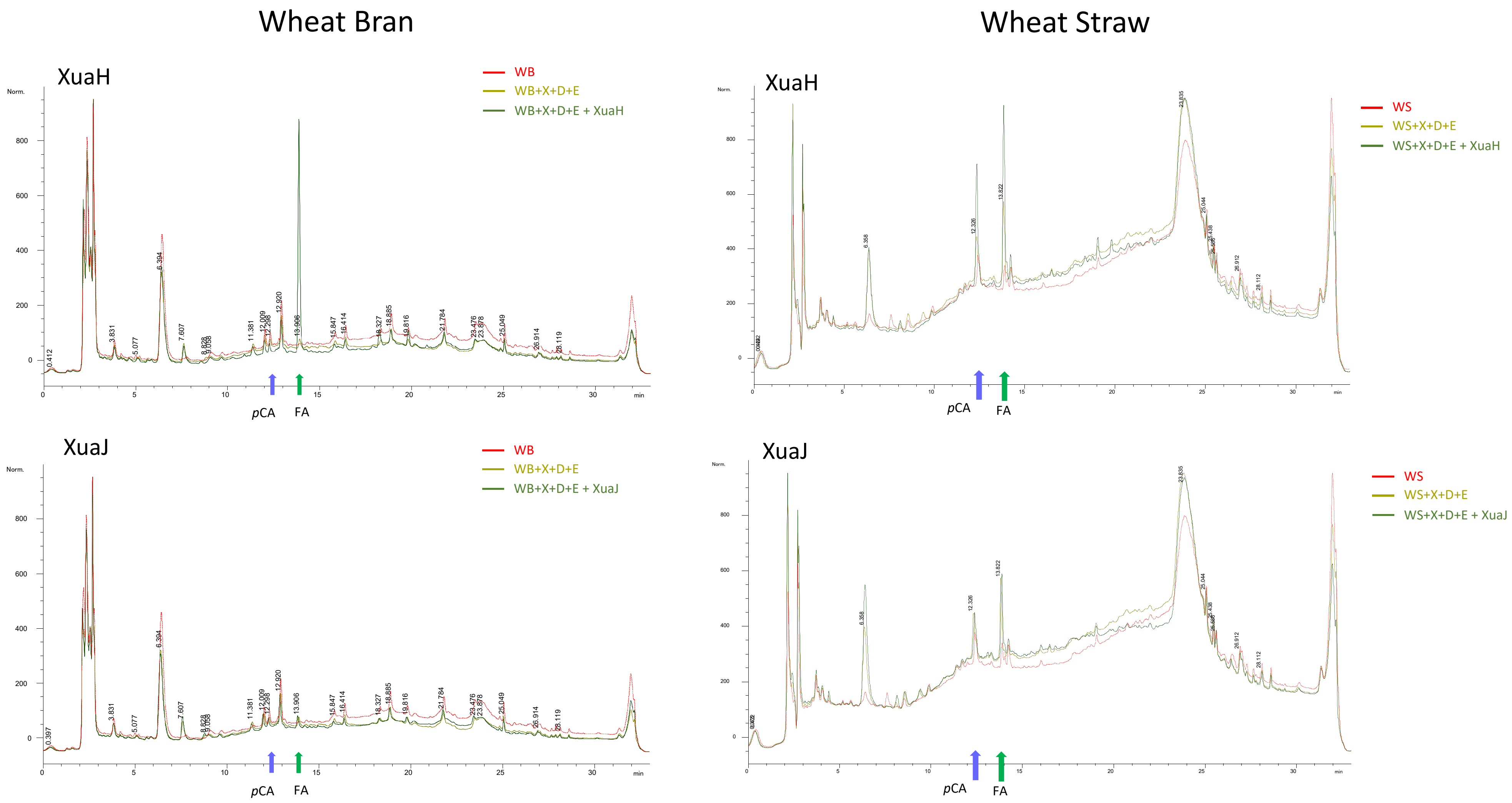

Wheat bran (WB) and wheat straw (WS) were pre-treated with a mixture of commercial xylanase (X) with the α-arabinofuranosidases XuaD and XuaE (X-D-E), with or without XuaH or XuaJ.

Figure S5. Overall view of the fold of the alphafold model of XuaH

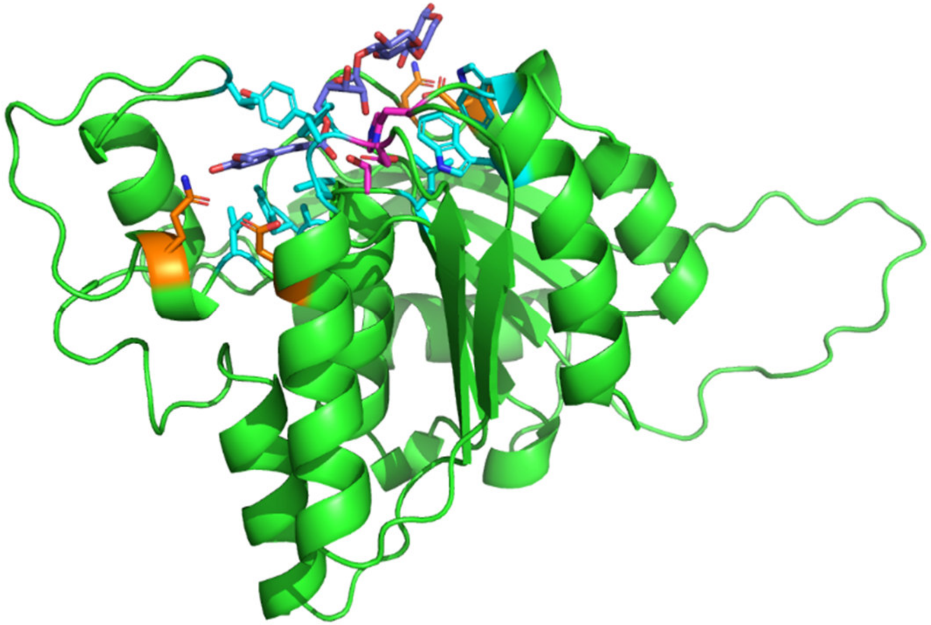

The alphafold model of XuaH is presented with the docked feruloyl-arabinoxylobiose (in blue). Residues of the catalytic triad including the nucleophile Ser127 are shown as sticks and highlighted in magenta. The other shown residues are the hydrophobic and hydrophilic amino acids of the active site cited in the text. They are highlighted in cyan and in orange, respectively.

Figure S6. Alignment of the primary sequence of XuaJ from *Ruminiclostridium cellulolyticum* with XacXaeA from *Xanthomonas citris* pv. citri str. 306 .

* indicates the catalytic triad amino-acids, S104, D508 and H510 in XuaJ and S115, D510 and H512 in XacXaeA.

```
                      10        20        30        40        50        60
                       |         |         |         |         |         |
XuaJxx0       -----------MGIILNS--NRPKIRLPRIISDGMVLQRNTDVKIWGWASPGEAVTVRFI
XacXaeA       MTASFVRRGCLLGLVLVSPCAWAVPTLPLLLADGAVLQRDQPMPVWGWSSPNAAIAVSFD
                         :*::* *    .   ** :::** ****:  : :***:**. *::* *
Prim.cons.    MTASFVRRGCL2G22L2SPC222222LP2222DG2VLQR222222WGW2SP22A22V2F2

                      70        80        90       100       110       120
                       |         |         |         |         |      *  |
XuaJxx0       GKAYHAFADDSGEWFVRLEQAKAGGPYDMYIETEESLEKITIKNILMGDVWLCSGQSNME
XacXaeA       GKRATVKADATGQWKVRLPAHAAGGPYVLRVQGDG--GELQVRDVLVGDVWLAGGQSNME
              **   . ** :*:* ***    ***** : :: :    :: ::::*:*****..******
Prim.cons.    GK22222AD22G2W2VRL2222AGGPY22222222SL22222222L2GDVWL22GQSNME

                     130       140       150       160       170       180
                       |         |         |         |         |         |
XuaJxx0       MKMDSVKDTYPDEIVHSCNDYIRHFLVPVKYDFEKPQTDLEAGIWEAANPESILDFTATG
XacXaeA       WPLAQASDG-PQAVAAANDAQLRQFKVPKSWSVQ-PQARLTGGEWKAATPANAGEFTAVG
                : ...*  *: :. : :  :*:* ** .:..: **: * .* *:**.* .  :***.*
Prim.cons.    2222222D2YP22222222222R2F2VP222222KPQ22L22G2W2AA2P22222FTA2G

                     190       200       210       220       230       240
                       |         |         |         |         |         |
XuaJxx0       YFFALKLFEKYNIPIGLINASLGGSPAEAWLSENALREFPEHYESAKQLSNRDYLDKVLR
XacXaeA       YFFAKELRASTGVPIGIVNSTWGGSAIEAWMDAASLGLNADQNQGAIEAIKQ--RDAAAQ
              **** :*  . .:***::*:: ***. ***:.  :*   .:: :.* :  ::   * . :
Prim.cons.    YFFA22L222222PIG22N222GGS22EAW22222L222222222A222222DY2D2222

                     250       260       270       280       290       300
                       |         |         |         |         |         |
XuaJxx0       EDQESAEAWYTALNQNDEGLKSNDIPFHDTEYDAPFWQNIKVPSYWEDEGVGNFNGVVWF
XacXaeA       AATGKRIARWPKV--EDEMPQWREAAFDDS--D---WDSIPVTKQWESSGYDGMDGIAWY
                  .  * :. :  :**  : .: .*.*:  *   *:.* *.. **..* ..::*:.*:
Prim.cons.    2222222A22222NQ2DE22222222F2D2EYDAPFW22I2V222WE22G22222G22W2

                     310       320       330       340       350       360
                       |         |         |         |         |         |
XuaJxx0       RKEIDIPSTLADKPARLVLGNIVDEDTAYINGIEVGTTPNQY-ITRKYSIQEGLLKEGKN
XacXaeA       RTTITLSAAEAKAGITLGVGQIDDSDTTYVNGQQVGSTEKQWNLPRVYQVPAAALKAGVN
              *. * :.:: *.    * :*:* *.**:*:** :**:* :*: :.* *.:  . ** * *
Prim.cons.    R22I222222A22222L22G2I2D2DT2Y2NG22VG2T22Q2N22R2Y222222LK2G2N

                     370       380       390       400       410       420
                       |         |         |         |         |         |
XuaJxx0       TIVLRVVNTAGKGGFYK-DKPYQLEIGDNIIELSGEWQYMIGAKSSPMPAPAFVQWRPLG
XacXaeA       HIAVRVEDLSGGGGMHGPDEQRFVQTSAGAKRALDGWKFRPAAVRVSLTD--NKNQLPTL
               *.:** : :* **::  *:   :: . .  .  . *::  .*   .:.     :  *
Prim.cons.    2I22RV2222G2GG222PD222222222222222222W22222A2222222PA22222P22

                     430       440       450       460       470       480
                       |         |         |         |         |         |
XuaJxx0       LYNGMIAPVTSYSIKGFIWYQGESNTKNPG--EYENLLKALIADWRQKWNMGNLPFLYVQ
XacXaeA       LYNQMIHPLQPFPVKGVIWYQGETNATDTGAVKYREQFAAMIRQWRAERGDKTLPFLWVQ
              *** ** *: .:.:**.******:*:.:.*  :*.: : *:* :** : .  .****:**
Prim.cons.    LYN2MI2P222222KG2IWYQGE2N2222GAV2Y22222A2I22WR2222222LPFL2VQ

                     490       500       510       520       530       540
                       |         |         |         |   * *   |         |
XuaJxx0       LPNFMEASEIPMESSWAELREAQRRTLSVPCTGMAVAIDLGEWNDIHPVNKKDVGFRLAL
XacXaeA       LANFKAGGDKGELSPWALLRESQSKTLALPATGQAVIIDIGNPTDIHPTNKRDVGHRLAL
              *.**  ..:    *.** ***:* :**::*.** ** **:*: .****.**:***.****
Prim.cons.    L2NF222222222S2WA2LRE2Q22TL22P2TG2AV2ID2G222DIHP2NK2DVG2RLAL

                     550       560       570       580       590       600
                       |         |         |         |         |         |
XuaJxx0       AAMKTAYRDKTIVAYGPMYQSGKIDGNRIILSFTDTGSGLTVKNGDRPEAFAIAGADRTF
XacXaeA       AARHVAYG-ETLVYSAPVFKRASFDGGKAVLGFDLQGSALQVRGGGAVQGFRIAGADQRF
              ** :.**  :*:*  .*::: ..:**.: :*.*   **.* *:.*.  :.* *****: *
Prim.cons.    AA222AY2D2T2V222P2222222DG2222L2F222GS2L2V22G22222F2IAGAD22F

                     610       620       630       640       650
                       |         |         |         |         |
XuaJxx0       VRADAEIIGDSVAVWSEKVSHPVYVKYAWADNPADANLYNLEGLPASPFTTE--
XacXaeA       HPATAQIDGDRVIVRSDAVAAPVAVRYGWSENPDDANLINRDALPVSPFRTDTW
                * *:* ** * * *: *: ** *:*.*::** **** * :.**.*** *:
Prim.cons.    22A2A2I2GD2V2V2S22V22PV2V2Y2W22NP2DANL2N222LP2SPF2T2TW
```

Table S1. Strains and vectors used in this study

Strain	Relevant characteristics	Source or reference
E.coli NEB5α	*fhuA2* Δ(*argF*-*lacZ*)*U*169 *phoA glnV*44 *Φ*80 Δ(*lacZ*)M15 *gyrA96* recA1 *relA1 endA1 thi-1 hsdR17*	New England Biolabs
E.coli BL21(DE3)	F^- *ompT hsdSB* (rB^-mB^-) *gal dcm* (DE3)	Invitrogen
R. cellulolyticum	Wild-type, H10, ATCC35319, DSM 5812	DSMZ
R. cellulolyticum MTL*xuaS*	*xuaS*::intron, Erm^r	This study
**Vector**	**Relevant characteristics**	**Source or reference**
pET22b(+)	*E. coli* expression vector, Amp^r	Novagen
pET28b(+)	*E. coli* expression vector, Km^r	Novagen
pET*xuaH*	pET22b(+) derivative carrying the NdeI-XhoI fragment encoding XuaH	[14]
pET*xuaI*	pET28b(+) derivative carrying the NcoI-XhoI fragment encoding XuaI	[14]
pET*xuaJ*	pET28b(+) derivative carrying the NcoI-XhoI fragment encoding XuaJ	[14]
pMTL007	*E. coli*/*Clostridium* shuttle vector (ColE1, pCB102)Ll.*ltrB* intron (*erm* BtdRAM2) under the control of P*fac*, *ltr* A; Cm^r/Tm^r	[28]
pMTL*xuaS*	pMTL007 derivative targeting *xuaS* (locus Ccel_1250)	This study
pSOSzeroTm	*E. coli*/*Clostridium* shuttle vector (ColE1, pIM13); Amp^r, Cm^r/Tm^r	[14]
pSOS*xuaSR*	pSOS956 *E. coli*/*Clostridium* shuttle vector (ColE1, pIM13); Amp^r, Cm^r/Tm^r, derivative, carrying the BamHI-EheI fragment encoding full length *xuaS* to *xuaR*, under the control of a weakened *Clostridium acetobutylicum* thiolase gene promotor	[14], this study
pBAD24	*E. coli* expression vector, Amp^r	[24]
pBAD*xuaR*	pBAD24 derivative carrying the NheI-SalI fragment encoding XuaR	This study
pUA66	*E coli* (pSC101), Km^r, *gfpmut2*	[24]
pUA66-*xua* IR1	pUA66 derivative carrying the 762 bp XhoI-BamHI intergenic region upstream of the gene at the locus Ccel_1250	This study
pUA66-xua IR2	pUA66 derivative carrying the 338 bp XhoI-BamHI intergenic region upstream of the gene at the locus Ccel_1252	This study
pUA66-xua IR3	pUA66 derivative carrying the 687 bp XhoI-BamHI intergenic region upstream of the gene at the locus Ccel_1255	This study
pUA66-xua IR4	pUA66 derivative carrying the 577 bp XhoI-BamHI intergenic region upstream of the gene at the locus Ccel_1256	This study
pUA66-xua IR5	pUA66 derivative carrying the 399 bp XhoI-BamHI intergenic region upstream of the gene at the locus Ccel_1259	This study
pUA66-xua IR6	pUA66 derivative carrying the 393 bp XhoI-BamHI intergenic region upstream of the gene at the locus Ccel_1260	This study
pUA66-xua IR7	pUA66 derivative carrying the 750 bp XhoI-BamHI intergenic region upstream of the gene at the locus Ccel_1261	This study

Amp^r, ampicilline resistance; Km^r, kanamycin resistance, Erm^r, erythromycin resistance; Cm^r/Tm^r, chloramphenicol/thiamphenicol resistance

Table S2. **Primers used in this study**

Name	Sequence	Note
xuaR 1251pBadNheD	AAAAGCTAGCAGGAGGAATTCACCATGTATAGGCTATTGATTGTAGATG	Reconstitution in *E. cc*
xuaR 1251pBadSalR_NEW	TTTTGTCGACTTATTTACCTGACAGCATGGTATC	
IR1pUA66XhoD	TTTTTCTCGAGTATCAGAATTTGCTGCTGAAGG	
IR1pUA66BamR	TTTTTGGATCCGGCTGAAAATACTGTTTCTCATC	
IR2pUA66XhoD	TTTTTCTCGAGGCCGTAGGGTATGAAACTG	
IR2pUA66BamR	TTTTTGGATCCTACAACGATTGAAGCTGTTAAAG	
IR3pUA66XhoD	TATATCTCGAGTTCTTCCAGGTGCAGTTC	
IR3pUA66BamR	GCGCAGGATCCACATCTACCCAGATGCTC	
IR4pUA66XhoD	AATATCTCGAGCTTCTAACGCCCACCTAC	
IR4pUA66BamR	GCGGCGGATCCCTTCCATTTCAATGTCATTATGAG	
IR5pUA66XhoD	CGCGGCTCGAGGTGTTAGGCGAATATAAGGC	
IR5pUA66BamR	GCGCTGGATCCCATACGCTCTGTACCTACAC	
IR6pUA66XhoD	CGCCGCTCGAGTGTAAATGAAACTCATGGTAATC	
IR6pUA66BamR	CCGCGGGATCCATCATTTGGTATCAAAGCATTA	
IR7pUA66XhoD	ATGCCCTCGAGCAATCATACGGATTGGTTGAC	
IR7pUA66BamR	ATATTGGATCCCCTCCAGACCGCCATC	
qPCR_16s_dir	CTATGTTTCTTGAGTGCCGG	qRT-PCR
qPCR_16s_rev	ATACTTATTGTGTTAACTCCGG	
qPCR-Ccel_1249-269 dir	CGCAATGTGCTTCTGCTTCA	
qPCR-Ccel_1249-547 rev	CCGCATCACCTGCTACATCA	
qPCR_*xuaS*_431_dir	CGGCAGGATTAAAGGGAGCA	
qRT_*xuaS*_708_rev	CCCATCACGATCCCGTTCTT	
qPCR_*xuaR*_277_dir	AAAGCAATTCAGCACCAGGG	
qPCR_*xuaR*_560_rev	CCAAGCAGCAGAATTACAGGA	
qPCR_*xuaA*_2nd_378_dir	ATCTGGAGAACTCCCTGATGT	
qPCR_*xuaA*_2nd_657_rev	AGTCTTAGGTGCCTGTAGGT	
qPCR_*xuaB*_346_dir	CCGCACTTTTTATCATGGGTTG	
qPCR_*xuaB*_584_rev	GCTGCTTCGTATAAACTGGGG	
qPCR_*xuaC*_429_dir	ATGGGCTTTGGTTCTTCCAG	
qPCR_*xuaC*_638_rev	CAGCTGTTCCAGTGACCTAC	
qPCR_*xuaD*_397_dir	CATGAAATGCAGCAGTGGGT	
qPCR_*xuaD*_647_rev	TCATCTACCGAAGCACCTCC	
qPCR_*xuaD'*_72_dir	AAGGGTAGTTAAATCGGGCG	
qPCR_*xuaD'*_254_rev	TACCACTTGTTGCCAGACG	
qPCR_*xuaE*_335_dir	GCGGTTGGGAACGTTATGTG	
qPCR_*xuaE*_619_rev	CAACACGCCTTCTGCTGTT	
qPCR_*xuaF*_293_dir	ACGAGGGAAAGTATCAGGGC	
qPCR_*xuaF*_524_rev	TCCCACATAGGTTCTCCACC	
qPCR_*xuaG*_297_dir	GCTGGGATTTATGCCGTCAC	
qPCR_*xuaG*_523_rev	ATTCGGACTCATCGGCATCC	
qPCR_*xuaH*_169_dir_2nd_	ACGGATTGGTTGACATGGAC	
qPCR_*xuaH*_403_rev_2nd	TTATTGCACCGTAACCACCC	
qPCR_*xuaI*_280_dir	GTAAAGGTGCTGAAGGCTGG	
qPCR_*xuaI*_560_rev	GACCGCATTGTTACATCCGA	
qPCR_*xuaJ*_222_dir_2nd	TGAAACGGAAGAGTCCCTTGA	
qPCR_xuaJ_447_rev_2nd	CTCCCAGATTCCTGCTTCCA	
qPCR-Ccel_1263-306 dir	ATACCGCAGGGTACTGGAAG	
qPCR-Ccel_1263-578 rev	TAGATTCCCGCACTGTTACCC	
xuaS-975a-EBS1d	CAGATTGTACAAATGTGGTGATAACAGATAAGTCTAAATCACTAACTTACCTTTCTTTGT	Targeted mutagenesi
xuaS-975a-IBS	AAAAAAGCTTATAATTATCCTTACACCTCTAAATCGTGCGCCCAGATAGGGTG	
xuaS-975a-EBS2	TGAACGCAAGTTTCTAATTTCGGTTAGGTGTCGATAGAGGAAAGTGTCT	
EBS universal primer	CGAAATTAGAAACTTGCGTTCAGTAAAC	
1250BamDir	AATTGGATCCTTCAAATAGAGAGGAAGTATAAAAATAAAATG	Complementation studi
1251NarRev	AATTGGCGCCTTATTTACCTGACAGCATGGTATC	
*xuaS*_593D_	TCAATACCTATGCGGGAAGC	Check *R. cellulolyticu* mutant strain
*xuaS*_1101R_	GCTTCAATTCGGCTTTCTGC	

Restriction sites are underlined (NheI GCTAGC; SalI GTCGAC; XhoI CTCGAG; BamHI GGATCC; NarI GGCGCC)
